# Supplementary material for: World’s human migration patterns in 2000–2019 unveiled by high-resolution data
Source: Nat Hum Behav. 2023 Sep 7;7(11):2023–37. doi: 10.1038/s41562-023-01689-4 (PMC10663150; doi:10.1038/s41562-023-01689-4)
Supplement: Supplementary file 1 — Supplementary Text, Figs. 1–6 and Tables 1 and 2. [file 41562_2023_1689_MOESM1_ESM.pdf]

nature human behaviour

Resource https://doi.org/10.1038/s41562-023-01689-4

World's human migration patterns in 2000–2019 unveiled by high-resolution data

In the format provided by the authors and unedited

Table of Contents

Supplementary text *2*

Socio-climatic bins 2

Rural-urban classification and net migration in rural and urban areas 2

Administrative zoning 3

Births and deaths 3

Downscaling births and deaths 3

Downscaling input variables 3

Regression model performance 3

Validation of the data 3

WorldPop 3

Births and deaths 3

Net migration 3

Supplementary Figures *5*

Supplementary Tables *11*

References *13*

Supplementary text

Error! Reference source not found.Socio-climatic bins

Socio-climatic bins were created by using global gridded data of aridity (Global Aridity Index(*1*)), human development (Human Development Index; downscaled by using methodology from Kummu et al(*2*)), and population counts for 2000–2019 from the WorldPop program(*3*). Our binning divides global inhabited areas into 100 socio-climatologically analogous zones, which have similar human development and climatic conditions. The binning was conducted in two steps. First, we divided all considered grid cells into 10 population-weighted quantiles based on HDI. After that, each HDI quantile was again divided into 10 population-weighted quantiles based on aridity. This division ensured that each bin incorporates 1% of the global population.

For both HDI and aridity, long-term averages were used. HDI data was provided for each year between 1990–2019 and averaged by taking a mean over 2000–2019. The Global Aridity Index and Potential Evapotranspiration defined by Trabucco and Zomer(*1*) provides a global estimate of aridity for 1970–2000 as a long-term average:

Aridity = MAP / MAET,

in which MAP refers to mean annual precipitation and MAET to mean annual evapotranspiration. The lower the aridity value, the higher the aridity, and the higher the aridity value, the higher the humidity. The original aridity raster with 30 arc-second resolution was aggregated to 5 arc-minute resolution prior to binning.

Extended Data Fig. 1 shows long-term averages of HDI, aridity and the derived socio-climatic bins. Extended Data Fig. 2 illustrates how the bins were divided.

Rural-urban classification and net migration in rural and urban areas

Extended Data Fig. 3 showcases an example of the urban extent data developed in this study. The data maps urban areas over two decades from 2000 to 2019. The data are provided in 5 arc-min resolution rasters, with grid cells classified as 1 (urban) or 0 (rural). Extended Data Fig. 3 shows the development and growth of urban areas in the South China Sea around the Malesian peninsula during three timesteps, as well as the global extent of urban areas in 2019.

Urban extent rasters were used to calculate net migration in urban and rural areas. Urban net migration was defined by multiplying a net migration raster with the urban extent raster for each year:

urban_migr_2000 = net_migr_2000 * urban_extent_2000

urban_migr_2001 = net_migr_2001* urban_extent_2001

...

urban_migr_2019 = net_migr_2019 * urban_extent_2019

Rural net migration was similarly derived for each year from net migration data by sub-setting all raster cells where the urban extent raster was valued 0 (i.e. defined as rural). Extended Data Fig. 4 illustrates gridded net migration in urban and rural areas for selected years (2000, 2010, and 2019).

The amount of urban and rural net migration at national, sub-national and communal scales for each year over 2000–2019 could be then calculated as a zonal sum over each administrative area.

Extended Data Fig. 5 illustrates cumulated (2000–2019) and aggregated urban and rural net migration at national, sub-national and communal scales.

Furthermore, we investigated how net migration was divided between rural and urban areas within each administrative area. We divided administrative areas into four classes depending on the "direction" of migration. In other words, if both urban and rural areas had net-positive migration then the admin region in question would be a 'net-receiver', whereas in the opposite case it would be a 'net-sender'. A case in which urban migration was net-positive and rural net-negative would be called 'urban pull – rural push' and the opposite would be called 'rural pull – urban push'.

Administrative zoning

The regional division used in the study is shown in Extended Data Fig. 6. The grouping is based on the United Nations (UN) country grouping(*4*).

Births and deaths

For births, we used two compiled databases, namely StatCompiler(*5*) and EUROSTAT(*6*), as well as national census data. For deaths, we used likewise two compiled databases, namely OECD(*7*) regional statistics and EUROSTAT(*8*), as well as national censuses. illustrates the origin of data used for each country – noting that for some countries, births and deaths data were available in different sources.

Downscaling births and deaths

Downscaling input variables

Extended Data Fig. 8 illustrates the spatial distribution of the downscaling input variables: HDI, population density, share of women of reproductive age (15–49), and share of life lived for an average person.

Regression model performance

We assessed the performance of regression models used to predict cell-wise birth and death rates, finding that the models predicted the birth and death rates well, with coefficient of determinations being 0.74 and 0.60, respectively (Table S1).

Validation of the data

Data developed in the study were validated against sub-national and national observations. Data used in the validation are described in Table S2.

WorldPop

WorldPop population grids were validated against OECD data. Results presented in Supplementary Figure 1 and Supplementary Table 2 show strong and significant ($p < 0.001$) correlation between observed (OECD) and modelled (WorldPop) values (Pearson's R=0.992-0.996, for all evaluated time steps: 2000, 2005, 2010, 2015, 2020).

Births and deaths

Gridded birth and death data were validated against EUROSTAT and OECD data, respectively. The results presented in Supplementary Figure 2 and Supplementary Table 2 show that the downscaled values were in line with reported values, the correlation coefficient (Pearson's R) being R=0.79 and R=0.76 for births and deaths with the significance being $p < 0.001$, respectively.

Net migration

Gridded net migration was validated against national and sub-national observations. Correlations between observed and modelled values were mainly strong (Pearson's R ranging between 0.61–0.9,

except for year 2020 when correlation was not statistically significant) and significant ($p<0.001$ in all years) at national level (Supplementary Figure 3). Largest differences between observed and modelled values were observed in 2019. This may be explained by the uncertainty in census data and reporting over the most recent years.

At the sub-national level, validation was done for the US, Europe and South Korea. US validation (Supplementary Figure 4) shows very strong and significant correlation between the modelled and observed data. For the US, the observed data was reported for different age groups as a 10-year cumulative sum. To obtain total net migration for the whole population, net migration in each age group was first summed together. Then, an accumulated sum over the reported years (2000–2010) was calculated from the here-developed data, and then compared to the reported values.

In Europe, the correlation between the modelled net migration and reported data is mainly strong and statistically significant, Pearson's R ranging between 0.31–0.88 ($p<0.001$) in years 2001, 2005, 2010, 2015 and 2020 (Supplementary Table 2). For 5- and 10-year cumulative net migration rates, correlations were moderate (0.3-0.57, $p<0.001$) (Supplementary Figure 6). For individual years, correlation between observed and estimated rates ranged between 0.17-0.49 (Supplementary Table 2). We suspect that the largest differences between the observed and modelled data are caused by differences in the definition of net migration. EUROSTAT defines net migration in two ways. Firstly, net migration is defined as the difference between in- and out-migration. Secondly, for some countries, the definition also includes a "statistical adjustment", meaning "other changes in the population figures between 1 January for two consecutive years which cannot be attributed to births, deaths, immigration or emigration." (*9*; metadata). We defined net migration as the difference between absolute and natural population change, thus, missing any detailed information of immigration or emigration and other flows in or out.

In South Korea, correlation (Pearson's R) ranges between 0.6–0.99 for the total and accumulated modelled and observed net migration counts in each year and year group (Supplementary Figure 5, Supplementary Table 2), respectively. Here, it should be noted that net migration counts for Sejong were provided only for 2012–2020 due to a district reform in 2012 in which Sejong was formed by joining parts of two other provinces (Jeongi-gunand and Chungcheongnam-do) (*10*). The reported net migration counts for Sejong were 1000-fold compared to our estimates and thus appeared as outliers. Thus, Sejong was excluded from the validation. The validation of migration rates shows strong and significant correlation for the five-year periods (2001-2005, 2006-2010) (Supplementary Figure 5a) and the first 10-year period of the century (2001–2010) (Supplementary Figure 5b). For other observed years and year groups, the correlation was weaker and insignificant.

Supplementary Figures

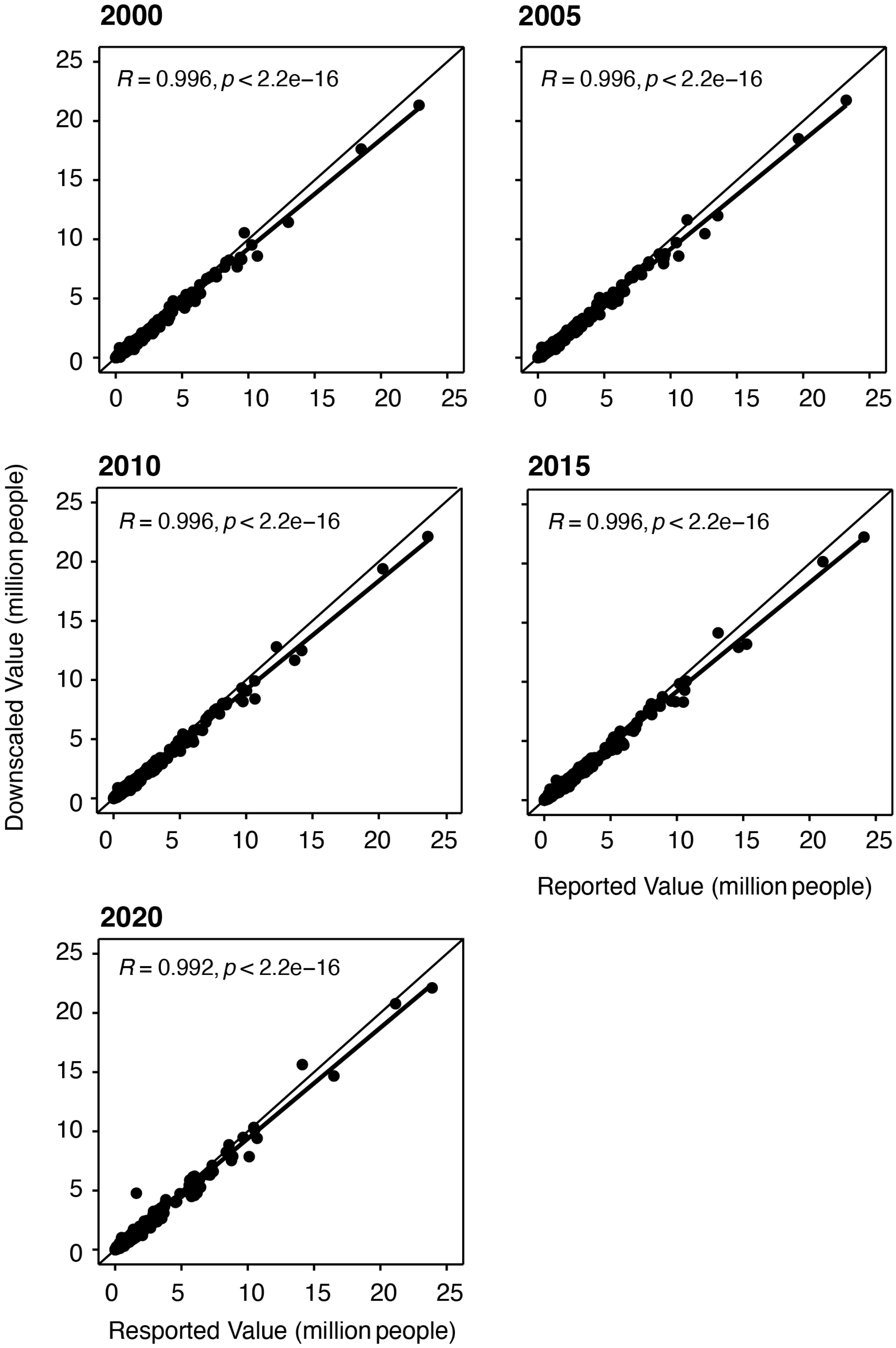

Supplementary Figure 1. Validation of the WorldPop data against reported data by OECD (*11*). Correlation was tested with Person correlation coefficient.

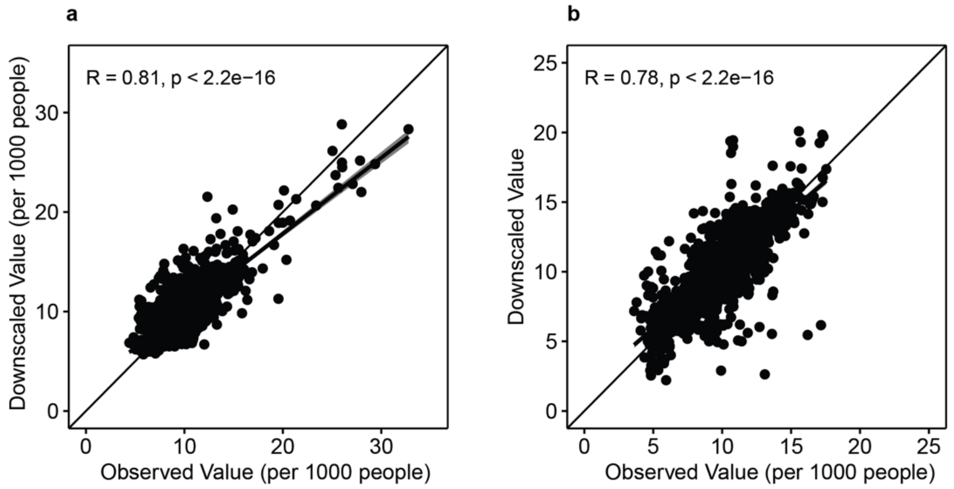

Supplementary Figure 2. Validation of (a) births against reported EUROSTAT data (*6*) and (b) deaths against reported OECD data (*7*). Correlation was tested with Person correlation coefficient.

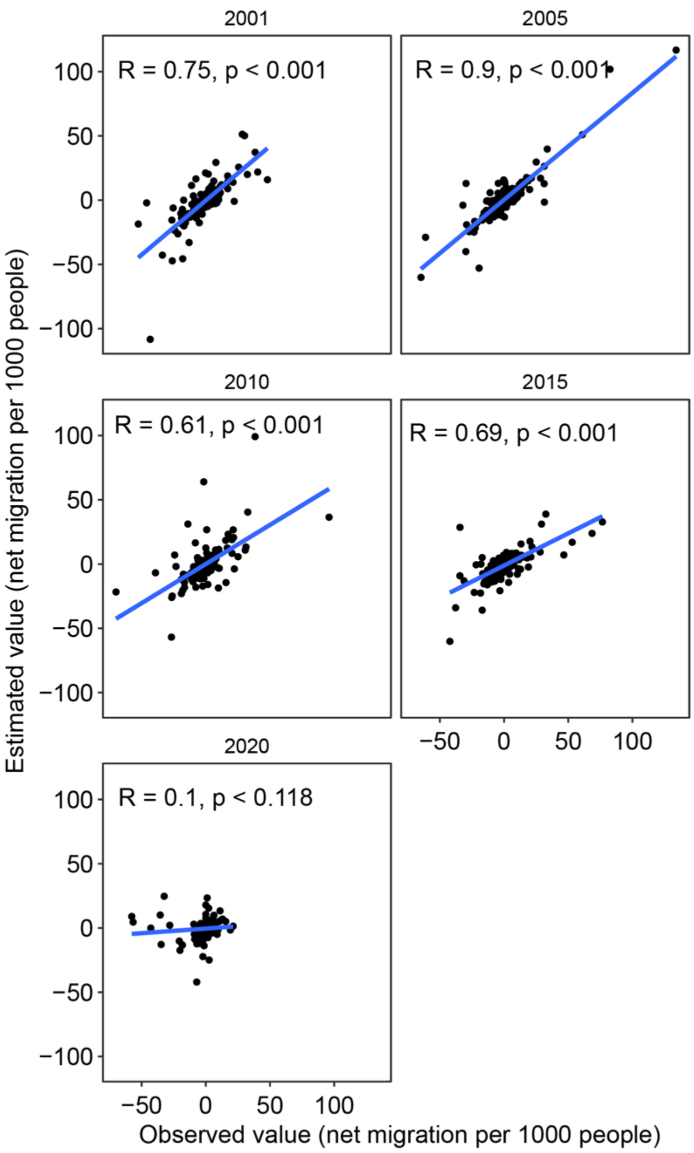

Supplementary Figure 3. National level validation of net migration data. Observed data provided by the UN (see source in Supplementary Table 2). Correlation was tested with Person correlation coefficient.

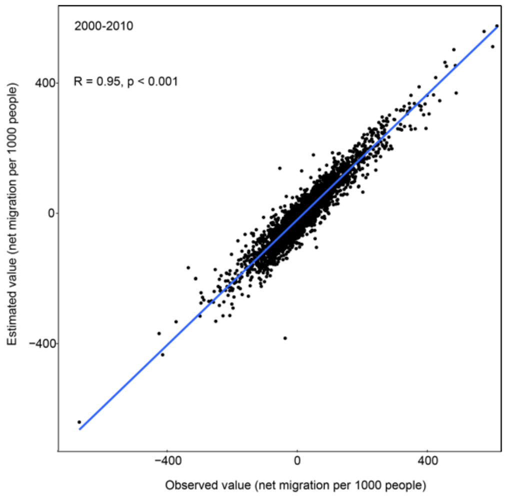

Supplementary Figure 4. Sub-national validation of the net migration rate (net migration per 1000 people) in the US counties over 2000–2010 (see source in Supplementary Table 2). Correlation was tested with Person correlation coefficient.

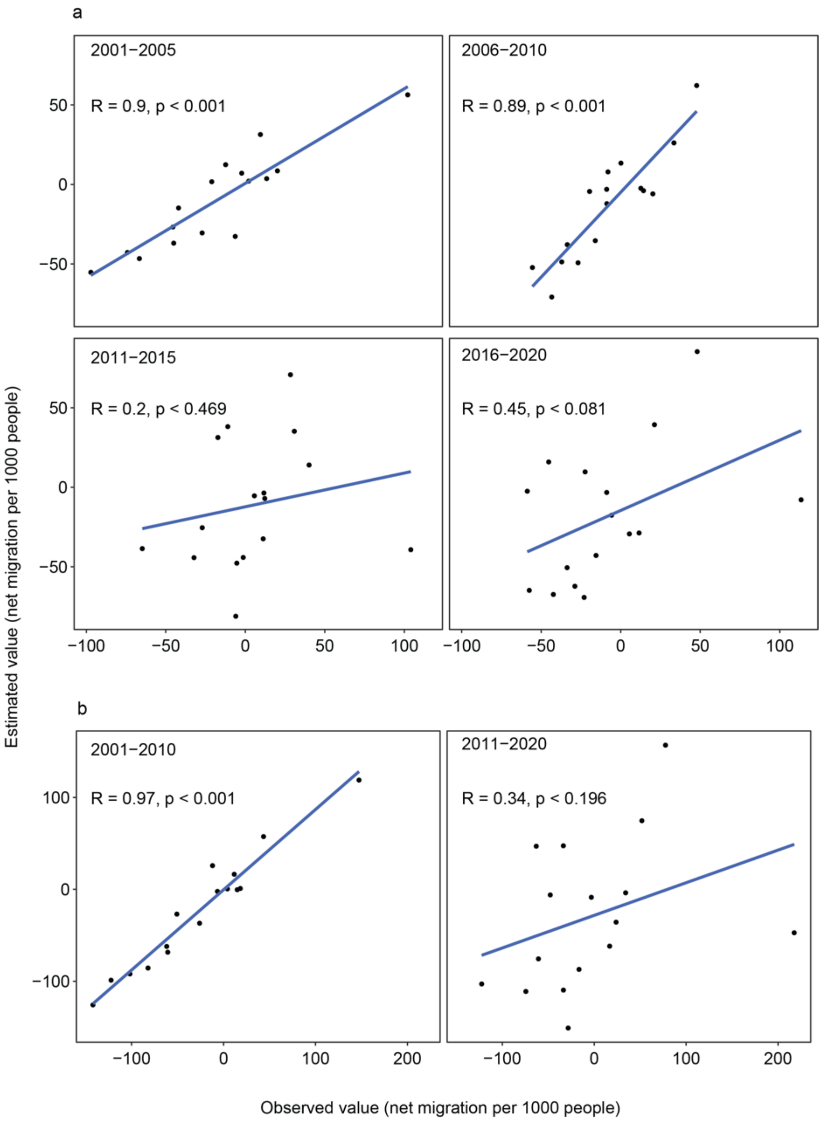

Supplementary Figure 5. Sub-national validation of (a) five-year and (b) ten-year cumulative sums of net migration rate (net migration per 1000 people) in South Korea's provinces (see source in Supplementary Table 2). Correlation was tested with Person correlation coefficient.

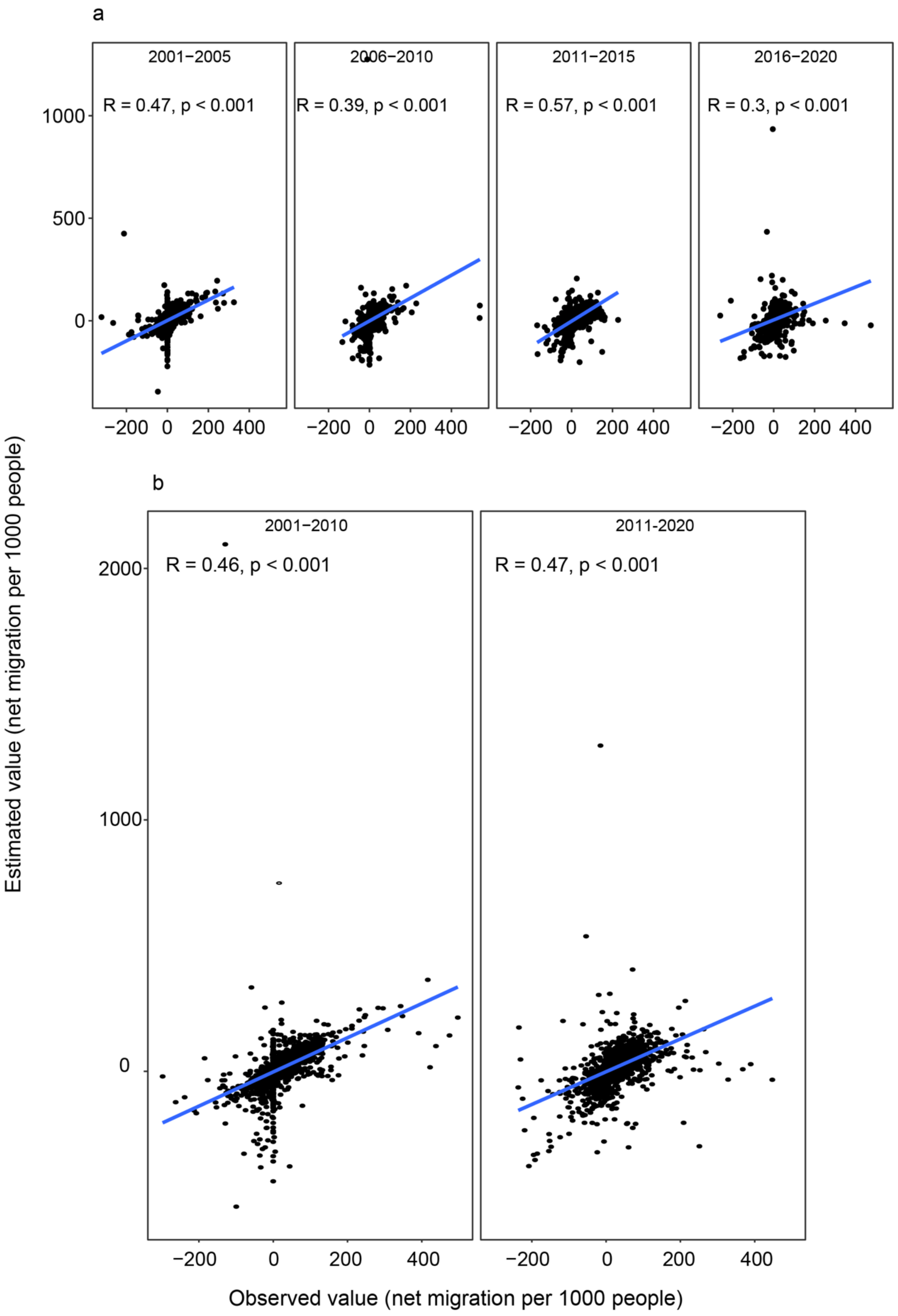

Supplementary Figure 6. Sub-national validation of (a) five-year and (b) ten-year cumulative net migration rates (net migration per 1000 people) in the EU (see source in Supplementary Table 2). Correlation was tested with Person correlation coefficient.

Supplementary Tables

Supplementary Table 1. Regression model performance to explain reported birth and death rates with selected independent variables (see Methods).

	R2	RMSE	Mean absolute error
Birth rate	0.74	5.84	4.52
Death rate	0.60	2.45	1.80

Supplementary Table 2. Datasets used for validation, spatial resolutions of those as well as number of observations and temporal extent and resolution. R represents Person's R.

Gridded data	Validation data	Spatial resolution	n	Temporal extent and resolution	R (only statistically significant results are presented, p < 0.001)	Reference
Births	EUROSTAT	Sub-national	1504	2001–2020	0.79	(*6*)
Deaths	OECD	Sub-national	1883	2001–2020	0.76	(*7*)
Population (WorldPop)	OECD	Sub-national	1818	2001, 2005, 2010, 2015, 2019	0.992-0.996	(*11*)
					**Net migration rate / Absolute net migration**	
Net migration	UN	National	238	2001, 2005, 2010, 2015, 2020	0.75; 0.9; 0.61; 0.69 / 0.83; 0.86; 0.72; 0.61;0.55	(*12*)
	EUROSTAT	Sub-national	1522	2001, 2005, 2010, 2015, 2020	0.24; 0.49; 0.41; 0.49; 0.17 / 0.59; 0.88; 0.72; 0.73; 0.31	(*9*)
				2001–2020 (5-year interval)	0.47; 0.39; 0.57; 0.3 / 0.69; 0.78; 0.76; 0.58	
				2001–2020 (10-year interval)	0.46; 0.47 / 0.77; 0.74	
	Korean Statistical Information Service KOSIS	Sub-national (South Korean provinces)	16	2001, 2005, 2010, 2015, 2019	0.6; 0.89; 0.79 / 0.96; 0.98; 0.95; 0.79; 0.98	(*13*)
				2001–2020 (5-year interval)	0.9; 0.89 / 0.99; 0.99; 0.79; 0.97	
				2001–2020 (10-year interval)	0.97 / 0.99; 0.92	
	Winkler et al.	Sub-national (US counties)	3056	2000–2010 (decadal)	0.95 / 0.98	(*14*)

References

1. A. Trabucco, R. Zomer J., Global Aridity Index and Potential Evapotranspiration (ET0) Climate Database v2. figshare. Fileset. https://doi.org/10.6084/m9.figshare.7504448.v3 (2019), (available at https://cgiarcsi.community).

2. M. Kummu, M. Taka, J. H. A. Guillaume, Gridded global datasets for Gross Domestic Product and Human Development Index over 1990–2015. *Scientific Data*. **5**, 180004 (2018).

3. WorldPop, www.worldpop.org - School of Geography and Environmental Science, University of Southampton; Department of Geography and Geosciences, University of Louisville; Departement de Geographie, Universite de Namur) and Center for International Earth Science Information Network (CIESIN), Columbia University (2018). Global High Resolution Population Denominators Project - Funded by The Bill and Melinda Gates Foundation (OPP1134076) https://dx.doi.org/10.5258/SOTON/WP00647 (2021), (available at https://dx.doi.org/10.5258/SOTON/WP00647).

4. Statistics Division of the United Nations Secretariat, Standard country or area codes for statistical use (M49) - Geographic Regions, (available at https://unstats.un.org/unsd/methodology/m49/). Accessed 02/2023. (2021).

5. The DHS Program, STATcompiler. Funded by USAID. http://www.statcompiler.com. [Accessed 02/2021], (available at https://www.statcompiler.com/en/).

6. EUROSTAT, “Live births and crude birth rate” (The statistical office of the European Union, Unit F2: Population and migration statistics) https://ec.europa.eu/eurostat/databrowser/ (accessed 02/2021) (2021), (available at https://ec.europa.eu/eurostat/web/products-datasets/-/tps00204).

7. OECD, Regional Statistics, Mortality crude rates by cause of death, large TL2 regions, small TL3 region. https://doi.org/10.1787/region-data-en. Accessed 02/2021 (2021).

8. EUROSTAT, “Deaths and crude death rate” (The statistical office of the European Union, Unit F2: Population and migration statistics) https://ec.europa.eu/eurostat/databrowser/ (accessed 02/2021) (2021), (available at https://ec.europa.eu/eurostat/web/products-datasets/-/tps00029).

9. EUROSTAT, “Population change - Demographic balance and crude rates at regional level (NUTS 3)” (Statistical Office of the European Union, Luxembourg, 2021), (available at https://ec.europa.eu/eurostat/databrowser/view/DEMO_R_GIND3/default/table?lang=en).

10. History of Sejong. *Sejong City: administrative capital de facto of Korea*, (available at https://www.sejong.go.kr/eng/sub01_0201.do).

11. OECD, Regional Statistics, Population by 5-year age groups, small regions TL3. https://doi.org/10.1787/region-data-en. Accessed 02/2021 (2021).

12. United Nations, Department of Economic and Social Affairs, Population Division, World Population Prospects 2022: Data for Net migration rate (per 1,000 population). https://data.un.org/. Accessed 02/2023. (2022).

13. KOSIS, Number of internal migrants for city, county, and district. Korean Statistical Information Database. https://kosis.kr/statHtml/statHtml.do?orgId=101&tblId=DT_1B26001_A01&conn_path=I2&language=en (accessed 02/2023) (2023).

14. Richelle Winkler, K. Johnson, C. Cheng, P. Voss, K. Curtis, County-Specific Net Migration by Five-Year Age Groups, Hispanic Origin, Race and Sex: 2000-2010: Version 1. Inter-university Consortium for Political and Social Research [distributor] (accessed 04/2022) (2013), , doi:10.3886/ICPSR34638.V1.
