# Supplementary material for: World’s human migration patterns in 2000–2019 unveiled by high-resolution data
Source: Nat Hum Behav. 2023 Sep 7;7(11):2023–37. doi: 10.1038/s41562-023-01689-4 (PMC10663150; doi:10.1038/s41562-023-01689-4)
Supplement: Supplementary file 2 — Reporting Summary [file 41562_2023_1689_MOESM2_ESM.pdf]

nature portfolio

Corresponding author(s): Matti Kummu

Last updated by author(s): Jun 6, 2023

Reporting Summary

Nature Portfolio wishes to improve the reproducibility of the work that we publish. This form provides structure for consistency and transparency in reporting. For further information on Nature Portfolio policies, see our Editorial Policies and the Editorial Policy Checklist.

Statistics

For all statistical analyses, confirm that the following items are present in the figure legend, table legend, main text, or Methods section.

n/a	Confirmed	
☐	☒	The exact sample size (*n*) for each experimental group/condition, given as a discrete number and unit of measurement
☒	☐	A statement on whether measurements were taken from distinct samples or whether the same sample was measured repeatedly
☐	☒	The statistical test(s) used AND whether they are one- or two-sided<br>*Only common tests should be described solely by name; describe more complex techniques in the Methods section.*
☒	☐	A description of all covariates tested
☐	☒	A description of any assumptions or corrections, such as tests of normality and adjustment for multiple comparisons
☒	☐	A full description of the statistical parameters including central tendency (e.g. means) or other basic estimates (e.g. regression coefficient) AND variation (e.g. standard deviation) or associated estimates of uncertainty (e.g. confidence intervals)
☐	☒	For null hypothesis testing, the test statistic (e.g. *F*, *t*, *r*) with confidence intervals, effect sizes, degrees of freedom and *P* value noted<br>*Give P values as exact values whenever suitable.*
☒	☐	For Bayesian analysis, information on the choice of priors and Markov chain Monte Carlo settings
☒	☐	For hierarchical and complex designs, identification of the appropriate level for tests and full reporting of outcomes
☒	☐	Estimates of effect sizes (e.g. Cohen's *d*, Pearson's *r*), indicating how they were calculated

Our web collection on statistics for biologists contains articles on many of the points above.

Software and code

Policy information about availability of computer code

Data collection	The data used in the analysis was collected manually from national censuses, demographic databases (StatCompiler and EuroStat) and other open data repositories. No software were used for data collection.
Data analysis	The analysis conducted in this paper was conducted with R (version 4.1.2) using R-Studio (RStudio 2021.09.0+351 "Ghost Orchid" Release). All R-scripts created for data processing, analysis and visualisation will be made openly available in GitHub upon publication.

For manuscripts utilizing custom algorithms or software that are central to the research but not yet described in published literature, software must be made available to editors and reviewers. We strongly encourage code deposition in a community repository (e.g. GitHub). See the Nature Portfolio guidelines for submitting code & software for further information.

Data

Policy information about availability of data

All manuscripts must include a data availability statement. This statement should provide the following information, where applicable:

- Accession codes, unique identifiers, or web links for publicly available datasets
- A description of any restrictions on data availability
- For clinical datasets or third party data, please ensure that the statement adheres to our policy

All the data used in this study are publicly available. The resulted datasets are available at the following open-access repository: http://doi.org/10.5281/zenodo.7997134, including the following datasets: annual global net-migration rates at grid scale as a multiband GeoTIFF with 5 arc-min resolution for 2000-2019;

annual global net-migration rates for adm0, adm1 and adm2 levels as polygon layers (gpkg-files); and annual global birth and death rates as multiband GeoTIFFs with 5 arc-min resolution for 2000-2019.
Data is visualised in online net-migration explorer at https://wdrg.aalto.fi/global-net-migration-explorer/. Data underlying the web application is available in the repository with all other data.

Human research participants

Policy information about studies involving human research participants and Sex and Gender in Research.

Reporting on sex and gender	N/A
Population characteristics	N/A
Recruitment	N/A
Ethics oversight	N/A

Note that full information on the approval of the study protocol must also be provided in the manuscript.

Field-specific reporting

Please select the one below that is the best fit for your research. If you are not sure, read the appropriate sections before making your selection.

☐ Life sciences ☒ Behavioural & social sciences ☐ Ecological, evolutionary & environmental sciences

For a reference copy of the document with all sections, see nature.com/documents/nr-reporting-summary-flat.pdf

Behavioural & social sciences study design

All studies must disclose on these points even when the disclosure is negative.

Study description

This study utilizes quantitative demographic, environmental and socio-economic data to 1) construct a novel quantitative gridded global dataset of human net-migration, and 2) conduct a quantitative analysis describing key features and underlying structures of the produced data in relation to several demographic, socio-economic and environmental factors.

Research sample

All data used in this study are openly available. The existing datasets for sub-national birth and death ratios are: national censuses, EuroStat database (1), StatCompiler (2), UN databases (3) and OECD databases (4). The origin of dataset for each country is specified in the Supplementary materials (Figure S8) provided in the submission.

Other datasets used include: aridity index (5), HDI (6&7), WorldPop 'population density' (8) and ‘Age and sex structures’ datasets (8).

1. EUROSTAT, “Live births and crude birth rate” (The statistical office of the European Union, Unit F2: Population and migration statistics, Luxembourg, 2021), (available at https://ec.europa.eu/eurostat/web/products-datasets/-/tps00204).
EUROSTAT, “Deaths and crude death rate” (The statistical office of the European Union, Unit F2: Population and migration statistics, Luxembourg, 2021), (available at https://ec.europa.eu/eurostat/web/products-datasets/-/tps00029).
2. The DHS Program, STATcompiler (https://www.statcompiler.com/en/).
3. Statistics Division of the United Nations Secretariat, Standard country or area codes for statistical use (M49) - Geographic Regions. (2021), (available at https://unstats.un.org/unsd/methodology/m49/).
4. OECD, Mortality crude rates by cause of death, large TL2 regions, small TL3 regions (2021).
5. A. Trabucco, R. Zomer J., Global Aridity Index and Potential Evapo-Transpiration (ET0) Climate Database v2 (2018), (available at https://cgiarcsi.community).
6. M. Kummu, M. Taka, J. H. A. Guillaume, Gridded global datasets for Gross Domestic Product and Human Development Index over 1990–2015. Scientific Data. 5, 180004 (2018).
7. J. Smits, I. Permanyer, The Subnational Human Development Database. Scientific Data 6, 190038 (2019).
8. WorldPop (www.worldpop.org - School of Geography and Environmental Science, University of Southampton; Department of Geography and Geosciences, University of Louisville; Departement de Geographie, Universite de Namur), Center for International Earth Science Information Network (CIESIN), Columbia University, Global High Resolution Population Denominators Project - Funded by The Bill and Melinda Gates Foundation (OPP1134076), (available at https://dx.doi.org/10.5258/SOTON/WP00647).

Data collection and processing are described in detail in the Methods.

Sampling strategy

We did do sampling in the analysis as we used all available data.

Data collection

Data for sub-national birth and death ratios were manually collected from several sources (see above), and compiled into one spreadsheet provided as supplementary material. Missing datapoints were filled with linear interpolation and extrapolation. The procedure is described in detail in the Methods section.

Timing	The data collected covers years 2000-2019.
Data exclusions	No data were excluded in the analysis.
Non-participation	The study solely used existing demographic data and did not engage individuals in data collection.
Randomization	We used all the available data and thus no randomization was needed.

Reporting for specific materials, systems and methods

We require information from authors about some types of materials, experimental systems and methods used in many studies. Here, indicate whether each material, system or method listed is relevant to your study. If you are not sure if a list item applies to your research, read the appropriate section before selecting a response.

Materials & experimental systems

n/a	Involved in the study
☒	☐ Antibodies
☒	☐ Eukaryotic cell lines
☒	☐ Palaeontology and archaeology
☒	☐ Animals and other organisms
☒	☐ Clinical data
☒	☐ Dual use research of concern

Methods

n/a	Involved in the study
☒	☐ ChIP-seq
☒	☐ Flow cytometry
☒	☐ MRI-based neuroimaging
